# Parental management of childhood fever and associated factors: progress and promise

**DOI:** 10.1016/j.jped.2026.101605

**Published:** 2026-09-04

**Authors:** Edward Purssell

**Affiliations:** a Anglia Ruskin University, Chelmsford, Essex, UK; b Little Havens Children’s Hospice, Benfleet, Essex, UK

The rational treatment of childhood fever remains one of the most challenging tasks facing parents and professionals alike. The study by Dias et al. in this journal shows both the challenges but also successes of rational evidence-based fever management [1]. The primary outcome was appropriate management of a febrile episode according to nine recommendations of the Brazilian Pediatric Society covering diagnosis and treatment [2]. This commentary will consider some of the issues around the treatment of fever pertinent to their findings, and highlight a few newer observations about temperature regulation. The use of antimicrobials is worthy of a review of its own, and so this will fall outside of the scope of this commentary.

The treatment of fever is an interesting mixture of old and new knowledge, with quite a lot that is still to be learned. Even though many people worry about fever, we know that fever is not, in itself harmful. However, in making this statement two important qualifications apply. Firstly, it is important to differentiate fever from hyperthermia; the first being a regulated rise in temperature, and the second overheating. The second qualification is to differentiate fever from its underlying cause. It is this latter issue that is most challenging when trying to establish the meaning of fever in children, because while fever may indicate a self-limiting (if distressing) illness, it may also be a symptom of more serious underlying disease. The word ‘fever’ is attached to a wide variety of different conditions, some of which are major causes of morbidity and mortality, perhaps explaining some of the anxiety attached to this word [3].

## What is a fever?

If fever is a raised body temperature, we must start with what constitutes a normal body temperature. As far back as 1944, Ivy identified three views of ‘normality’: the normative view where a person meets a given standard, for example a temperature of 37 °C; the clinical or pathological view which allows for temperature to be higher or lower than this as long as the child is not significantly ill; and the statistical view using some measure of variation from the mean such as ± 2 standard deviations which would include around 95% of children [4].

Definitions of fever that involve a single normative vale such as 37.5 °C are necessarily arbitrary as they don’t take individual or diurnal variation, or measurement route into account. One set of values that meets Ivy’s statistical view sets a normal range for axilla temperatures in children at 34.7 °C to 37.3 °C, equating to an oral range of 35.5 °C to 37.5 °C, and for the ear 35.8 °C to 38 °C [5]. These are both relatively easy approaches as they use set values, the pathological approach which moves beyond simple numbers towards individual assessment is more challenging.

In this study the normative cut-off for defining fever was equal or over 37.5 °C when taken in the axilla. Axillary temperatures are probably the safest and most straightforward measurement method as it can be used with all ages. However, because of poor agreement between different methods of measurement and possible operator error, individual readings should be interpreted with awareness of the probability of error and in the context of a complete and holistic assessment of child and family. Although parents may have a preference of a particular measurement method, many will adopt whatever method is recommended [6].

## The physiology of fever

The methods by which body temperature is regulated and fever is achieved are becoming clearer even if not completely understood. The concept of the hypothalamic thermostat often found in textbooks and papers is not particularity helpful in understanding this. The thermostat analogy suggests that fever is controlled by a switch that can be adjusted at will, when in reality it is far more complex than this as the body does not have a single temperature, but rather different temperatures at different sites. One model suggests that rather than being regulated by a unified single controller, temperature regulation is comprised of a number of independent thermo-effector loops comprised of afferent and efferent divisions [7]. The main role of the hypothalamus is to receive and integrate thermal information from around the body via warm and cold-sensitive neurons, and to activate responses to regulate this. Under normal circumstances the human body is a heat generating machine, needing to losing heat mainly through radiation and evaporation, as well as convection and conduction, and certainly not needing to generate more. Thus, additional heat producing or conserving mechanisms such as non-shivering thermogenesis and shivering are tonically inhibited through the action of warm sensitive gamma-aminobutyric acid (GABA) GABAergic neurons.

Fever results from the presence of endogenous or exogenous pyrogens including cytokines such as IL-1 and exogenous substances such as bacterial lipopolysaccharide (LPS). Binding of these substances to appropriate cellular receptors leads to the release of arachidonic acid from the cell membrane by the action of phospholipase A_2_ and its subsequent conversion to prostaglandin E2 (PGE_2_) by cyclooxygenase (COX) enzyme COX-2 [8]. PGE_2_ in turn binds to EP3 receptors (EP3R), most of which are linked to G_i_ inhibitory G-proteins found on warm sensitive neurons resulting in a reduction of intracellular cAMP. Stimulatory EP3γ receptors are found on cold sensitive neurons [9].

Although EP3 bearing cells are found throughout in many regions of the brain, the key area seems to be the preoptic area of the hypothalamus (POA). This area of the brain integrates cool and warm sensory information from peripheral thermoreceptors, local warm signals, and pyrogenic signals transmitted by PGE_2_. Under normal circumstances additional heat generating and conserving mechanisms are tonically or continuously inhibited by GABAergic activity. However, when PGE_2_ binds to POA EP3R warm sensitive neurons their GABAergic action is itself inhibited causing disinhibition of heat generation and hat conservation mechanisms leading to the increase in temperature that we know as fever [10].

## Overheating

One anxiety related to fever is that left untreated temperature may continue to rise. Fever’s ‘glass ceiling’ of 41 °C–42 °C has been noted as the temperature beyond which fever does not extend. This likely results from a mixture of endogenous antipyretics and warm sensitive neurons in the POA which together prevent overheating in fever [9,11]. Therefore, while not an inherent problem for the febrile child, hyperthermia and heat stroke is a potential problem. The key is to balance the issues of over and under wrapping to avoid overheating and cold-related discomfort respectively.

## Febrile seizures

The causes of febrile seizures are likely to be multifactorial, including: neuronal inflammation affecting the balance of neuronal excitation and inhibition; changes in brain pH possibly caused by respiratory alkalosis; and genetic factors including mutations in Na+ ion channels SCN1A and SCN2A and GABA_A_ γ2 among others [12]. In particular increased levels of IL-1β in the central nervous system may cause an imbalance between the excitatory neurotransmitter glutamate and the inhibitory GABA [13].

A systematic review and meta-analysis looking at the use of antipyretics for the prevention of febrile seizure recurrence found a total of 8 studies. Of the three RCTs, one looked at recurrence in the same episode of fever and found some effect from the use of paracetamol, the odds ratio (OR) for recurrence being 0.33 (95% CI 0.19 to 0.57) but with a low certainty of evidence. Two RCTs out of a total of 4 studies looking at recurrence in distant fever episodes found no such effect from antipyretics, the OR being 0.92 (0.57 to 1.48) with a moderate certainty of evidence [14].

## Rigors

Rigors, which are characterized by shivering and a feeling of being cold, are a feature of some febrile episodes. The mechanism by which this occurs is becoming clearer and consists of two parts: the first is cold sensory information in addition to PGE_2_ in the POA leading to shivering, vasoconstriction and non-shivering thermogenesis; and the second is similar signals being sent to the central nucleus of the amygdala (CeA) which results in increased cold sensitivity, chills, and warmth seeking behavior. The combination of shivering and chills leading to what we commonly refer to as a rigor [15]. Seen from this perspective rigors while unpleasant and distressing to observe are really just an adaptation of the body and an indication of a need for warmth. Understanding this dual mechanism: the physiological effects mediated through the POA, and the limbic emotional response via the CeA goes some way to explaining why physical methods such as tepid sponging don’t work and can cause discomfort [16].

## The rational use of antipyretics

The rationale for drug use is variously described as being to reduce discomfort [2,17] and distress [18]. The difference in terminology is probably not important; although exactly what is meant by these terms is perhaps less clear. All of the drugs that are recommended in either of the three guidelines have analgesic in addition to their antipyretic properties and many conditions that lead to fever might also reasonably cause pain. Perhaps more controversially they may also have the effect of reducing parental anxiety.

While most guidelines recommend against using antipyretic drugs with the sole purpose of reducing temperature, there is some variation in the recommended antipyretic drugs and perhaps most interestingly in recommendations regarding alternating drugs. While Brazilian [2] and Italian [17] guidance recommends against such a practice, British guidance permits this if distress persists or recurs before the next dose of a single drug is due [18]. The authors note that despite most guidelines seemingly not recommending alternating drugs this remains a common practice. The British guidance is perhaps not helpful here and should be updated to reflect the majority of expert opinion.

## Dosing

In this study three drugs were recommended for use in the treatment of fever: dipyrone, paracetamol, or ibuprofen without combination. All of the drugs recommended for the treatment of fever or fever-related distress or discomfort related to fever are safe at recommended doses, with paracetamol and ibuprofen having similar safety profiles [19]. However, overdoses and toxicity, particularly related to paracetamol can occur even when given at apparently therapeutic doses [20] and the possibility of other toxicities is always present. In particular age-banded dosing for drugs could result in significant over and under-dosing of children who are light or heavy for their age, and so where possible the weight-based approaches recommended in this study would appear to be safer. Although overdosage seems more dangerous, repeated sub-therapeutic doses can have a cumulative effect.

## Conclusions

There are many questions still to be answered. The exact benefit associated with fever is still to be elucidated, as are those children most at risk or who are likely to get greatest benefit from fever. While there is an emerging international consensus about many aspects of the treatment of fever, some differences do still exist. The ongoing and widespread nature of the fear of fever and remains a challenge. Despite many years of education, numerous papers and official guidance fear and non-evidence-based treatments persist. This study suggests that this is the case in Brazil as it is in so many other places. Why this is the case, and what can be done to achieve long-term change are questions of significance.

One possibility is that fear of fever, just like fever itself, is an adaptive response to alert parents and carers to potentially serious infections. According to this hypothesis, some anxiety is adaptive if it results in parental and professional action, although this has implications for health providers.

As the indication for pharmacological treatment of fever is primarily distress or discomfort, being clear about the role of these medicines is important. Reframing conversations towards their role in relieving pain, distress and discomfort might be helpful, and guidance on this subject should emphasize this more holistic approach including non-pharmacological interventions. Finally, and perhaps more controversially, a positive effect of the safe and limited use of antipyretics on reducing parental and professional anxiety may be that this reduction is transmitted to the child, leading to a beneficial circle of reduced fear and anxiety.

## Data availability

Not applicable.

## Conflicts of interest

The author declares no conflicts of interest.
